# Surveillance of COVID-19 vaccine effectiveness: a real-time case–control study in southern Sweden

**DOI:** 10.1017/S0950268822000425

**Published:** 2022-03-02

**Authors:** Jonas Björk, Carl Bonander, Mahnaz Moghaddassi, Magnus Rasmussen, Ulf Malmqvist, Fredrik Kahn, Malin Inghammar

**Affiliations:** 1 Clinical Studies Sweden, Forum South, Skåne University Hospital, Lund, Sweden; 2 Division of Occupational and Environmental Medicine, Lund University, Lund, Sweden; 3 School of Public Health and Community Medicine, Institute of Medicine, University of Gothenburg, Gothenburg, Sweden; 4 Social Medicine and Global Health, Department of Clinical Sciences Malmö, Lund University, Malmö, Sweden; 5 Department of Clinical Sciences Lund, Section for Infection Medicine, Skåne University Hospital, Lund University, Lund, Sweden

**Keywords:** COVID-19 vaccines, epidemiologic methods, epidemiological monitoring, vaccine effectiveness

## Abstract

The extensive register infrastructure available for coronavirus disease 2019 surveillance in Scania county, Sweden, makes it possible to classify individual cases with respect to hospitalisation and disease severity, stratify on time since last dose and demographic factors, account for prior infection and extract data for population controls automatically. In the present study, we developed a case–control sampling design to surveil vaccine effectiveness (VE) in this ethnically and socioeconomically diverse population with more than 1.3 million inhabitants. The first surveillance results show that estimated VE against hospitalisation and severe disease 0–3 months after the last dose remained stable during the study period, but waned markedly 6 months after the last dose in persons aged 65 years or over.

## Introduction

Vaccines are since their introduction in December 2020 the primary mitigation strategy to combat coronavirus disease 2019 (COVID-19). The emergence of new severe acute respiratory syndrome coronavirus 2 (SARS-CoV-2) variants with higher risk of transmission (i.e. variants of concern, VOC) has stressed the importance of continuously surveil COVID-19 vaccine effectiveness (VE) and waning immunity [1–4]. The population and health care registers in Sweden and the other Nordic countries contain extensive individual-level data for all residents that can be cross-referenced [5]. Such register infrastructures offer excellent opportunities for detailed epidemiologic surveillance but are currently underused. The present study aimed to investigate how the Swedish register infrastructure can be used to surveil COVID-19 VE in real time. To this end, we developed an automatic case–control sampling design using data with complete population coverage from Scania (Skåne), an ethnically and socioeconomically diverse region in southern Sweden exceeding 1.3 million inhabitants.

## Methods

### Study design

wThe study cohort included all persons residing in Skåne, Sweden, on 27 December 2020 (baseline; *n* = 1 384 530) when vaccinations started [6], and was followed for 315 days until 7 November 2021. Individuals who died or moved out from the region were censored on the date of death or relocation, yielding 1 353 488 persons remaining in the cohort at the end of the follow-up.

The first to be vaccinated in Sweden were nursing home residents, their caregivers and frontline health care workers, followed by the general population in age groups in descending order, currently down to age 12. Three different vaccines have been used in Sweden: BNT16b2 mRNA (Comirnaty, Pfizer-BioNTech), mRNA-1273 (Spikevax, Moderna) and ChAdOx1-SARS-CoV-2 (Vaxzevria, AstraZeneca). The timing of the second dose depended on vaccine type and schedule but was given in median 42 days after the first. From 1 September 2021, a third booster dose was offered, starting with nursing home residents, older people and immunocompromised.

### Data sources

The different data sources were linked using the personal identification number assigned to all Swedish residents at birth or immigration [7]. Individual-level data on country of birth, civil status, residency and vital status were obtained from the Swedish Total Population Register. Weekly updates on vaccination date, type of vaccine and dose were obtained from the National Vaccination Register, and data on positive SARS-CoV-2 test results from the electronic system SMINet, both kept at the Public Health Agency of Sweden. Data from regional registers and electronic health records were accessed continuously. Hospitalisations 5 days before until 14 days after a positive SARS-CoV-2 test result and U07.1 (ICD-10) among the diagnoses were regarded as caused by COVID-19. Severe disease among the hospitalised was defined as a need of oxygen supply ≥5 l/min or admittance to an intensive care unit (ICU).

### Case–control sampling

To avoid conflation of varying infection pressure over time in the population with waning VE, we used continuous density case–control sampling [8] nested within the study cohort described above. A case was defined as a person with a first-time positive test or any positive test more than 12 weeks after a prior positive test. For each case, 10 controls without a positive test the same week as the case or 12 weeks prior were randomly selected from the complete study cohort, matched with respect to sex and age (5-year groups: 0–4, 5–9, 10–14, …, 95–99 and 100+).

### Statistical analysis

We used conditional logistic regression (Stata SE 14.2, Stata Corp, command *clogit*) for the 1:10 case:control matched sets to estimate the odds ratio (OR) and VE = 1 − OR together with 95% confidence interval (CI, accounting for individual clustering in the full-period estimates) for the association between vaccination status (at least two *vs*. zero doses) and risk of infection (positive test), being hospitalised or developing severe COVID-19. Only doses received at least 7 days before the case date were counted within each matched set when vaccination status was assessed. Vaccination status among those with at least two doses was grouped according to time since last dose (0–3, 3–6 or >6 months) and vaccine type. Results were (besides the matching) presented unadjusted, but prior infection was included in a sensitivity analysis.

## Results

### Descriptive results

VE was monitored weekly, from week 10 (8 March, 71 days after vaccination start) to week 44 in 2021 (7 November, 315 days after vaccination start). In total, 52 125 COVID-19 cases occurred during this period, of whom 1492 (2.9%) were hospitalised and 611 (1.2%) classified as severe (oxygen supply ≥5 l/min or ICU admittance). The weekly infection rate varied between 297 (week 18) and down to 13 (week 27) per 100 000 persons. Routine sequencing of samples of infected cases in the study region showed that the SARS-CoV-2 alpha variant (B.1.1.7) was the dominating VOC until week 26 after which the majority of cases were delta (B.1.617.2) [9]. The identified cases were considerably younger on average towards the end *vs.* at the start of the follow-up (Table 1). BNT16b2 mRNA (Pfizer-BioNTech) was the dominant vaccine type with 79% of all administrated doses during the follow-up.

**Table 1.** Characteristics (%) of the COVID-19 cases and sex and age matched controls, stratified by the follow-up period for monitoring of VE. Percentages have been calculated separately for cases and controls in each period

	Follow-up period, year 2021					
	Period 1, weeks 10–20 (days 71–147)		Period 2, weeks 21–32 (days 148–231)		Period 3, weeks 33–44 (days 232–315)	
	Cases	Controls	Cases	Controls	Cases	Controls
*N*	38 223	382 230	5 875	58 750	8027	80 270
Age						
0–17	18.1	18.4	18.8	20.7	30.1	29.2
18–39	36.8	36.5	49.4	47.5	34.9	35.8
40–64	38.4	38.4	28.2	28.2	28.7	28.7
65	6.7	6.7	3.6	3.6	6.3	6.3
Sex						
Females	49.8	49.8	50.3	50.3	52.0	52.0
Males	50.2	50.2	49.7	49.7	48.0	48.0
Born abroad	22.3	26.0	25.0	26.0	37.6	24.4
Civil status						
Married	35.7	32.1	28.7	25.4	31.5	26.7
Widow/widower	1.2	1.2	0.9	0.7	1.5	1.3
Divorced	8.4	9.1	6.8	6.4	8.4	7.0
Single	54.7	57.7	63.7	67.5	58.6	65.0
Vaccine doses						
0	94.7	90.9	77.9	70.2	64.0	44.0
1	4.4	5.7	15.9	18.1	8.9	11.4
2	1.0	3.4	6.2	11.7	26.9	44.4
3					0.2	0.1
Vaccine type^a^	*N* = 365	*N* = 13 123	*N* = 366	*N* = 6876	*N* = 2174	*N* = 35 791
Pfizer	92.3	90.3	72.1	72.5	81.0	79.1
Moderna	6.3	8.5	10.4	11.7	6.6	13.0
AZ	1.1	0.8	12.0	7.9	8.7	5.0
Mixed	0.3	0.5	5.5	7.9	3.8	2.9
Time since last dose^a^						
0–3 months	89.9	92.4	69.7	77.2	50.0	66.3
3–6 months	10.1	7.6	27.9	21.6	36.3	27.4
≥6 months	0.0	0.0	2.5	1.2	13.7	6.3
Prior SARS-CoV-2 infection	0.6	7.4	2.7	11.1	2.6	12.9
Hospitalised	3.0	–	1.9	–	3.0	–

a Only persons with at least two doses.

### Surveillance results

**Fig. 1.**
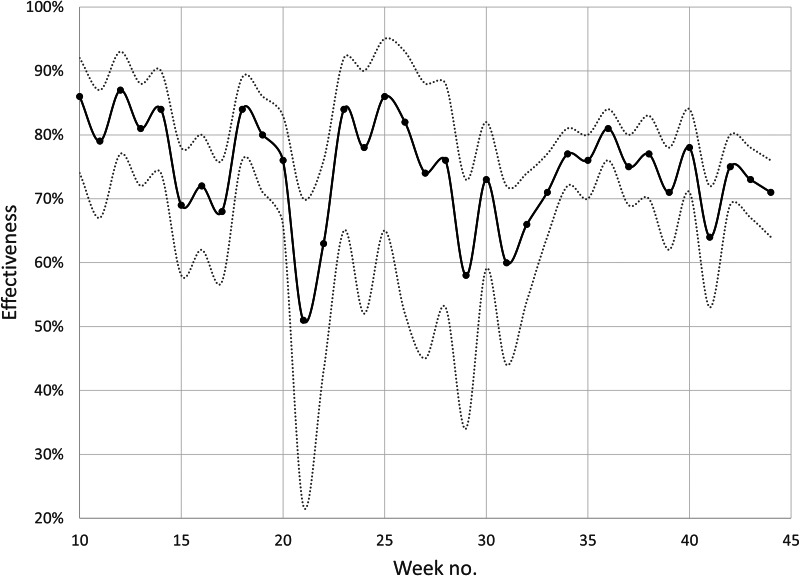
Weekly surveillance in Scania county, southern Sweden, during 2021 weeks 10–44 of the estimated effectiveness against SARS-CoV-2 infection after at least two doses of any of three COVID-19 vaccines. Grey dotted lines represent 95% CIs.

**Fig. 2.**
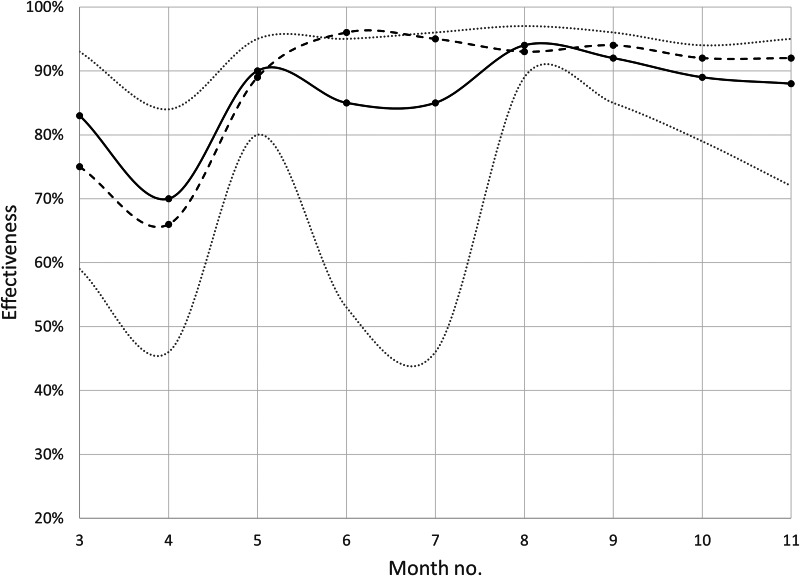
Monthly surveillance in Scania county, southern Sweden, during March–November 2021 (including week 44) of the estimated effectiveness against COVID-19 hospitalisation (solid black curve) and severe disease (oxygen supply ≥5 l/min or ICU admittance; dotted black curve) after at least two doses of any of three vaccines. Grey dotted lines represent 95% CIs for effectiveness against hospitalisation.

The estimated effectiveness against infection after at least two doses of any of three vaccines was 76% in median, and the weekly estimates varied between 51% and 87% with no evident time trend (Fig. 1). At least two doses of any of the three vaccines offered high protection against hospitalisation (median effectiveness 87%, range 70–94%) and severe disease (median effectiveness 93%, range 66–96%), here aggregated monthly due to small numbers (Fig. 2). Estimated effectiveness against infection (Supplementary Fig. S1A) and hospitalisation (Supplementary Fig. S1B) 0–3 months after the last dose remained stable during the study period. Waning effectiveness against hospitalisation was consistently noted 6 months after the last dose, but with substantial fluctuations due to the small numbers.

### Average effectiveness against infection, hospitalisation and severe disease

**Fig. 3.**
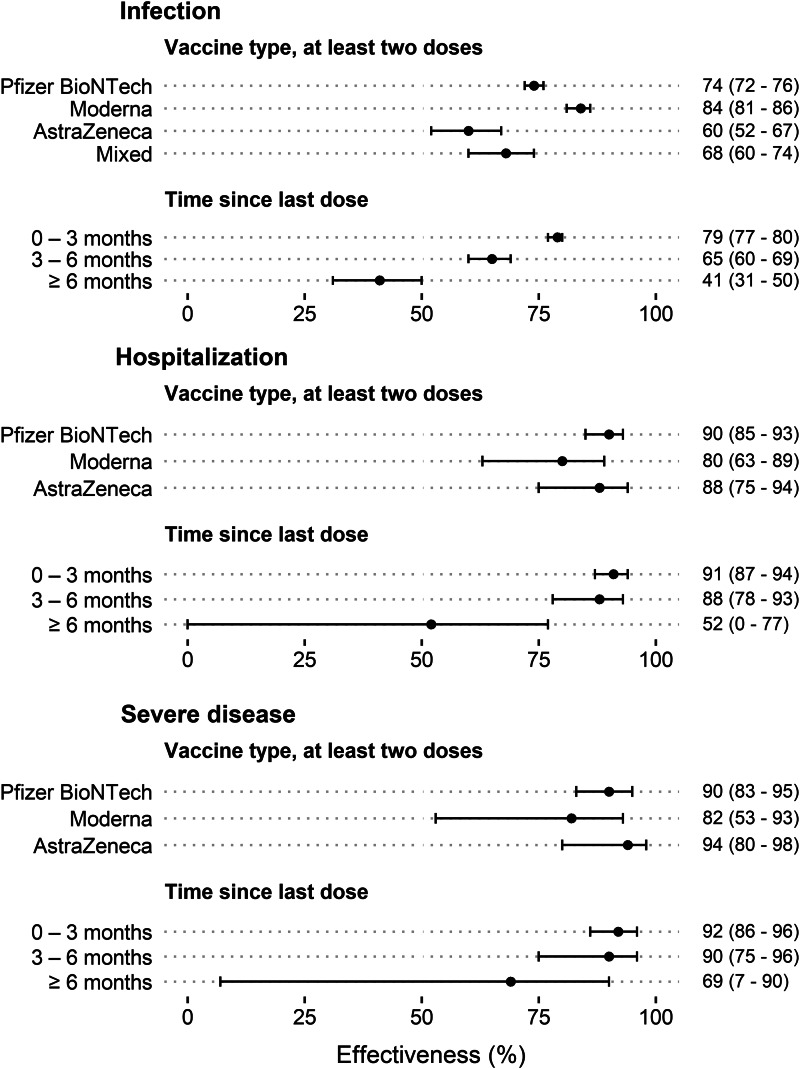
Average effectiveness (per cent; 95% cluster-robust CIs) of the COVID-19 vaccination during 2021 weeks 10–44 in protecting infection, hospitalisation and severe disease (oxygen supply ≥5 l/min or ICU admittance) in relation to vaccine type and time since last dose.

The mRNA vaccines (Pfizer-BioNTech and Moderna) exhibited higher effectiveness on average against infection than the vector vaccine (AstraZeneca; Fig. 3). All three vaccines offered strong protection against hospitalisation and severe disease both overall and specifically in individuals ≥65 years (Fig. 3 and Supplementary Fig. S2). VE waned considerably with time since last dose, especially in individuals ≥65 years. The protection against hospitalisation and severe disease remained more satisfactory in younger individuals but was also more statistically uncertain (Supplementary Fig. S2). Prior SARS-CoV-2 infection offered strong protection against new infection (average effectiveness 87%, 95% CI 86–88%) and hospitalisation (average effectiveness 90%, 95% CI 80–95%, not in tables), but did not confound the estimates of VE.

## Discussion

The extensive register data available in our real-time surveillance system allow us to classify hospitalised cases further concerning disease severity, stratify by time since last dose and demographic factors, account for prior SARS-CoV-2 infection and extract data for population controls automatically. The density case–control sampling is a convenient approach to avoid bias from underlying time trends in the population [8]. Such bias could occur if, e.g. associations between age or time since last dose among the vaccinated and the infection pressure on society are not considered during design or analysis. Selective COVID-19 testing depending on the vaccination status could still bias the case–control sample but is unlikely to impact estimated effectiveness against hospitalisation and severe disease.

The surveillance was based on data with complete population coverage, but was nevertheless hampered by limited population size and a low infection rate during parts of the study period. As a comparison, the population in Scania county is only 1/7 of the Israeli population, a well-known example where continuous surveillance of COVID-19 VE is conducted [3]. However, after adaptions, our extensive surveillance system should be possible to implement at the national level in Sweden, with a population exceeding 10 million.

Our initial surveillance result presented here show more marked waning VE among persons aged 65 years or over 6 months after the last dose. This finding is not consistent with results from the Israeli population where a decrease in effectiveness of similar magnitude in all age groups has been observed [3]. Differences in design and how the varying infection pressure in society over time is handled may be one explanation for the diverging results. Consistent with our finding, recent immunological investigations observed a more decreased immune humoral response among older people and nursing home residents and also among males and persons with immunosuppression [10–12]. It is for this reason important to continue the surveillance in order to study the effects of additional booster doses in specific groups as well as in the population more broadly, and to monitor protection against VOC [13].

The present investigation demonstrates the strength of combining individual-level population and health care register data to monitor VE in real time. A natural extension of the surveillance system would be to add further individual-level data on socioeconomic conditions, disease histories and care needs among older people, all available in the Swedish register infrastructure. Such additions would make it possible to monitor protection in vulnerable populations separately as a basis for decisions on additional booster doses, campaigns to increase vaccine uptakes in subgroups or other directed interventions.

## Data Availability

Aggregated surveillance data from the present study are publicly available at https://sodrasjukvardsregionen.se/kliniskastudier/covid-vacciner-skyddseffekt/.

## Supplementary material

For supplementary material accompanying this paper visit https://doi.org/10.1017/S0950268822000425. click here to view supplementary material
