# Supplementary material for: Surveillance of COVID-19 vaccine effectiveness: a real-time case–control study in southern Sweden
Source: Epidemiol Infect. 2022 Mar 2;150:e59. doi: 10.1017/S0950268822000425 (PMC8924553; doi:10.1017/S0950268822000425)

## Supplementary material

**Supplementary Figure 1.** Monthly surveillance in Scania county, Southern Sweden, during March - November 2021 (including week 44) of the estimated effectiveness against **A)** SARS-CoV-2 infection, **B)** COVID-19 hospitalization. Solid curves represent 0-3, dotted curves 3-6 and dashed curves more than 6 months since last dose of any of three vaccines.

**A.**


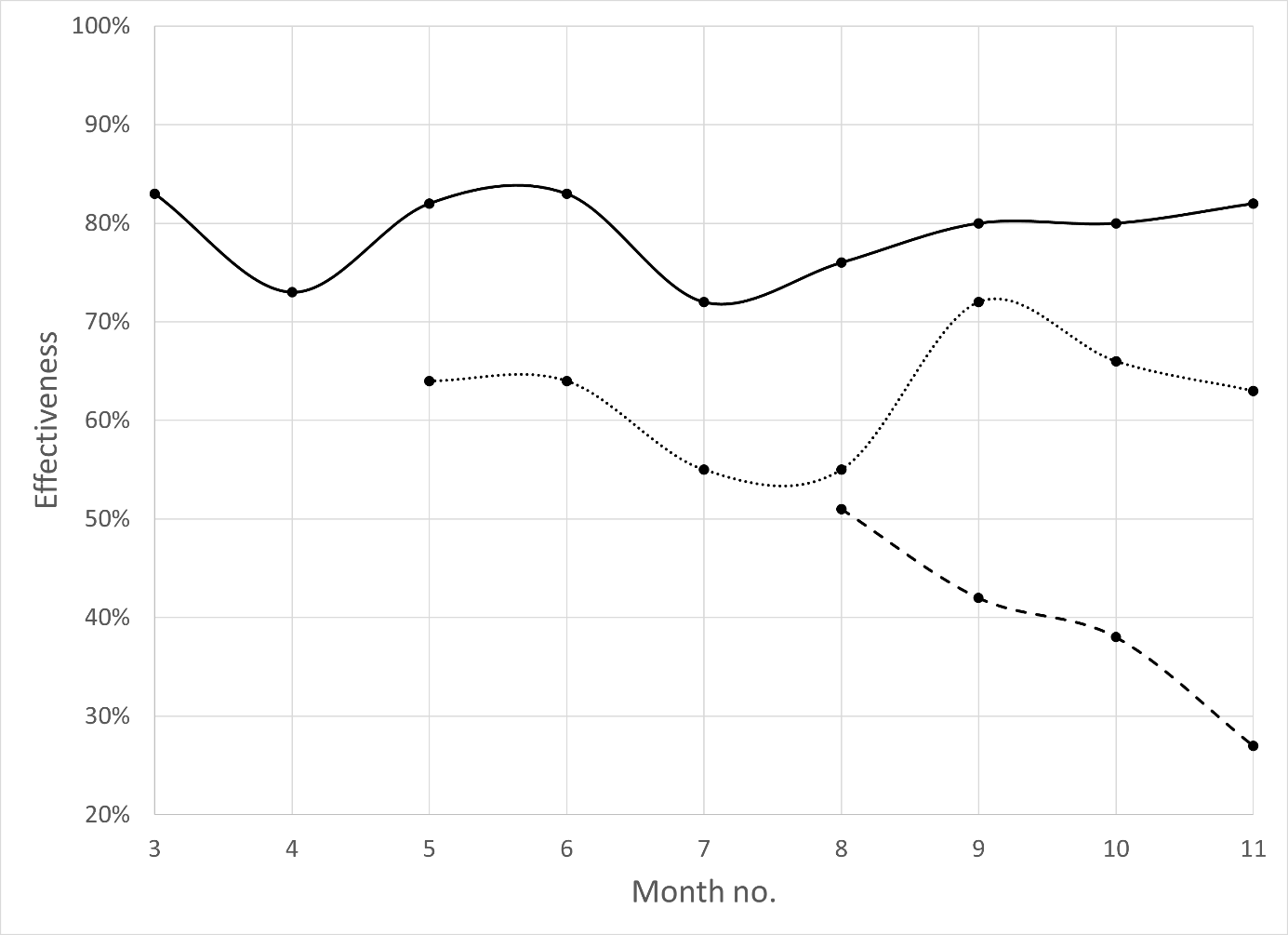


**B.**

**
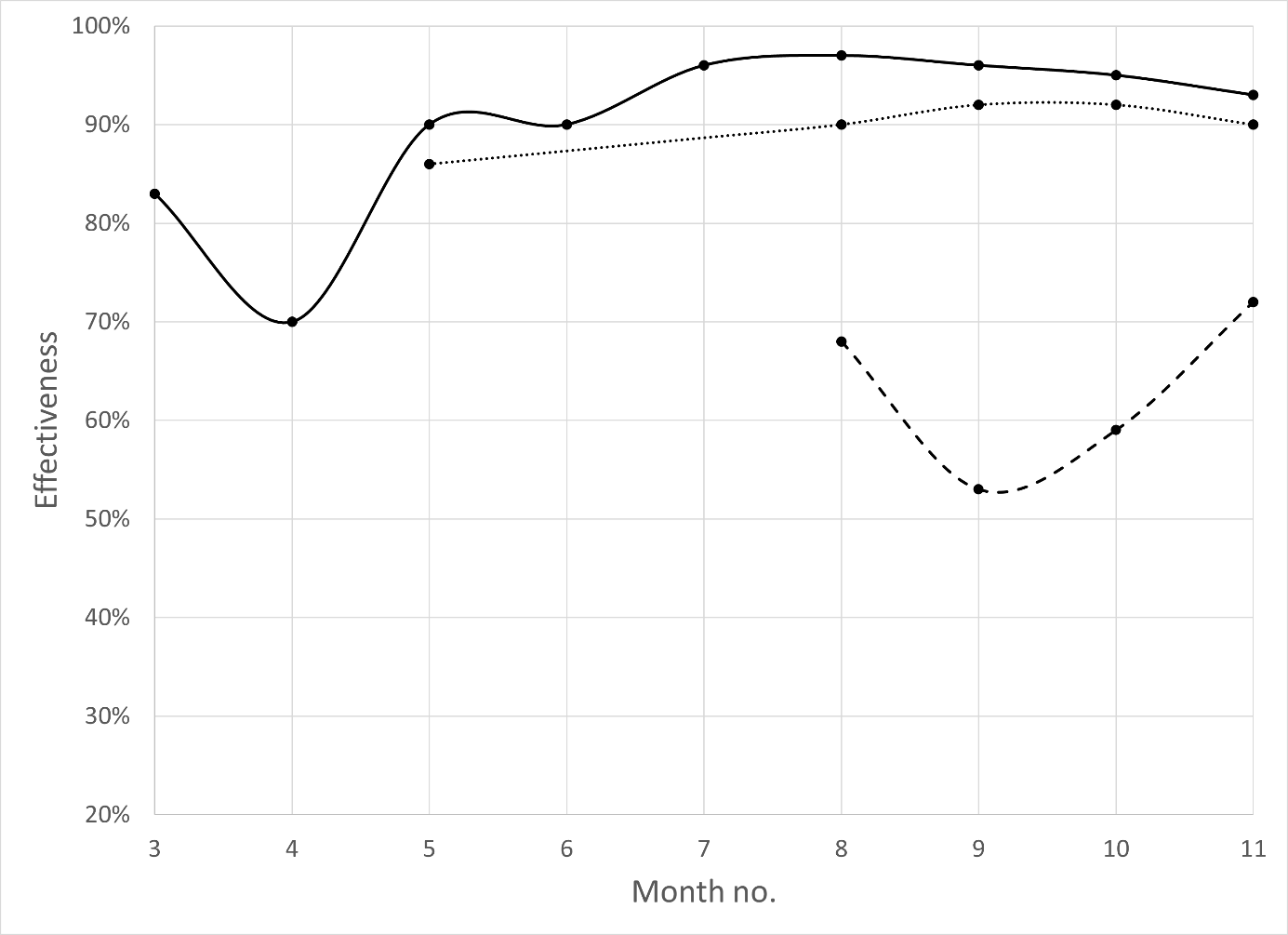
**

Note that the dashed curve (3-6 months since last dose) lacks reliable estimates for June – July 2021 (month 6 – 7) due to small numbers.

**Supplementary Figure 2.** Average effectiveness (percent; 95% cluster-robust CIs) of the COVID-19 vaccination during week 10 – 44 2021 in protecting infection, hospitalization and severe disease (oxygen supply ≥ 5 L/min or ICU admittance) in relation to vaccine type and time since last dose, stratified by age.


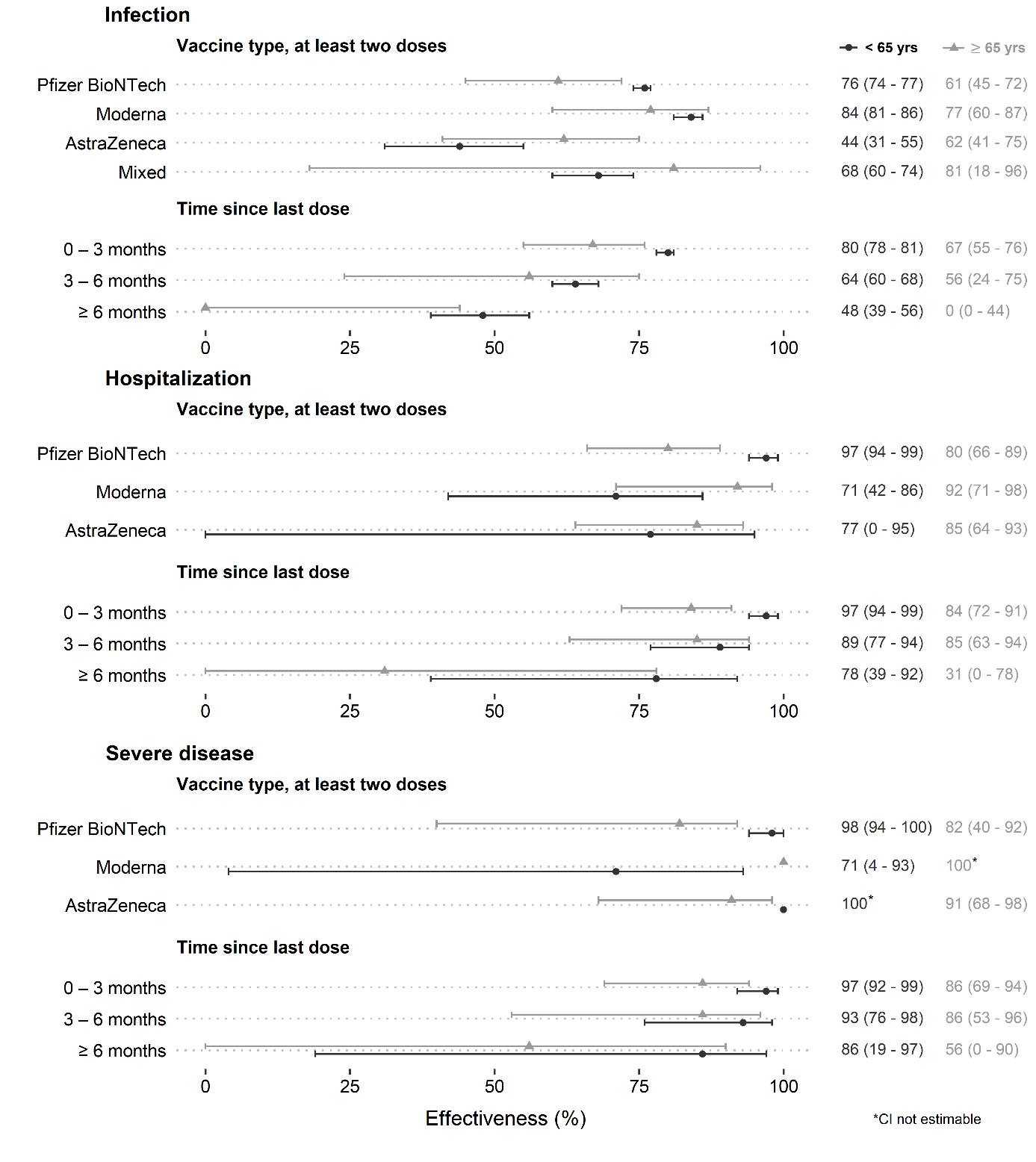

Supplement: Supplementary file 1 [file S0950268822000425sup001.docx]
